# Fusion of the BCL9 HD2 domain to E1A increases the cytopathic effect of an oncolytic adenovirus that targets colon cancer cells

**DOI:** 10.1186/1471-2407-6-236

**Published:** 2006-10-04

**Authors:** Christophe Fuerer, Krisztian Homicsko, Alexander N Lukashev, Anne-Laure Pittet, Richard D Iggo

**Affiliations:** 1 NCCR Molecular Oncology Programme, Swiss Institute for Experimental Cancer Research (ISREC), Epalinges, Switzerland; 2 Current address: Bute Medical School, University of St Andrews, Fife KY16 9TS, Scotland, UK

## Abstract

**Background:**

The Wnt signaling pathway is activated by mutations in the APC and β-catenin genes in many types of human cancer. β-catenin is stabilized by these mutations and activates transcription in part by acting as a bridge between Tcf/LEF proteins and the HD2 domain of the BCL9 coactivator. We have previously described oncolytic adenoviruses with binding sites for Tcf/LEF transcription factors inserted into the early viral promoters. These viruses replicate selectively in cells with activation of the Wnt pathway. To increase the activity of these viruses we have fused the viral transactivator E1A to the BCL9 HD2 domain.

**Methods:**

Luciferase assays, co-immunoprecipitation and Western blotting, immunofluorescent cell staining and cytopathic effect assays were used to characterize the E1A-HD2 fusion protein and virus in vitro. Growth curves of subcutaneous SW620 colon cancer xenografts were used to characterize the virus in vivo.

**Results:**

The E1A-HD2 fusion protein binds to β-catenin in vivo and activates a Tcf-regulated luciferase reporter better than wild-type E1A in cells with activated Wnt signaling. Expression of the E1A-HD2 protein promotes nuclear import of β-catenin, mediated by the strong nuclear localization signal in E1A. Tcf-regulated viruses expressing the fusion protein show increased expression of viral proteins and a five-fold increase in cytopathic effect (CPE) in colorectal cancer cell lines. There was no change in viral protein expression or CPE in HeLa cells, indicating that E1A-HD2 viruses retain selectivity for cells with activation of the Wnt signaling pathway. Despite increasing the cytopathic effect of the virus in vitro, fusion of the HD2 domain to E1A did not increase the burst size of the virus in vitro or the anti-tumor effect of the virus in an SW620 xenograft model in vivo.

**Conclusion:**

Despite an increase in the nuclear pool of β-catenin, the effects on viral activity in colon cancer cells were small, suggesting that factors acting downstream of β-catenin are limiting for viral replication and toxicity in these cells. The approach of fusing E1A to a protein domain implicated in oncogenic signaling could be used to selectively increase the activity of oncolytic viruses targeting several other pathways defective in cancer.

## Background

The Wnt signaling pathway is pathologically activated in many different types of human cancer [1]. Mutations in either the APC or β-catenin genes are seen in about 90% of human colorectal cancers [1]. Most APC mutations truncate the protein N-terminal to the axin binding domain. This prevents the assembly of the multiprotein complex that normally phosphorylates the N-terminus of β-catenin in the absence of Wnt ligand [2]. In hereditary non-polyposis colon cancer (HNPCC), APC is wild type but β-catenin is mutated in its N-terminal phosphorylation sites. When phosphorylated on these sites β-catenin is targeted for proteasomal degradation by the β-TRCP ubiquitin ligase [3]. Unphosphorylated β-catenin is stable and translocates to the nucleus, where it binds to members of the Tcf/Lef family of transcription factors and recruits coactivators such as p300, BCL9 and pygopus to activate transcription of Wnt target genes [4-7]. BCL9 is the human homolog of the Drosophila legless gene. A protein containing only the HD2 domain of BCL9 and the NHD domain of pygopus is able to rescue the embryonic phenotype of legless and pygopus mutant flies [4]. Since the HD2 domain binds to β-catenin, this suggests that the main function of BCL9 is to act as an adaptor that brings together β-catenin and pygopus.

During an adenoviral infection, E1A is the first gene to be expressed by the virus. Two main spliced forms are produced, 12S and 13S, which encode proteins differing in the presence of a zinc finger domain (Conserved Region 3, CR3) that supplies the major transcription activation function in E1A. The CR3 domain binds to ATF proteins, transcription factors that bind to cognate sites in the early viral promoters, and to the Sur2 subunit of the Mediator complex [8-10]. We have previously described replicating adenoviruses with Tcf sites in the early promoters [11,12]. These viruses replicate selectively in cells with activation of the Wnt signaling pathway, including many colorectal cancer cells. Since the Tcf sites replace the ATF sites in the viral promoters, the transactivation function of E1A is entirely replaced by Tcf-dependent coactivator functions in these viruses. In some colorectal cancer cell lines, the level of Tcf-dependent transactivation is insufficient to achieve wild type virus replication [12]. To circumvent this problem, we have fused E1A to the BCL9 HD2 domain in a Tcf-regulated virus. We show that E1A-HD2 proteins are able to bind to β-catenin and can activate transcription from Tcf-regulated promoters better than wild type E1A. Despite showing five-fold more activity in cytopathic effect assays in vitro, the activity of the virus was unchanged in xenografts of SW620 colon cancer cells in vivo.

## Methods

### E1A-HD2 mutagenesis

To create an E1A-HD2 fusion gene, an XmaI restriction site was inserted at the C-terminus of the E1A 12S coding sequence by PCR with the primers CGGGCCTGGGGCGTTTACAG (oCF83) and GGTAAGGTGTAAACCTGTGAT TGC (oCF84) to give the plasmid pALP3. The HD2 sequence was amplified from the BCL9 cDNA with the primers TACCCGGGAGCAATAGCTCTTCAG (oCF85) and ACCCCGGGATTCTGCTGCGGTCCCCCAC (oCF86). The product was cut with XmaI (underlined) and inserted into the XmaI site of pALP3 to give pALP5. The E1A 12S-HD2 sequence was back-cloned into pCF9 [12] with HpaI and BamHI to give pALP9, a pCDNA3 vector expressing the E1A 12S-HD2 fusion protein. The E1A-HD2 sequence was transferred from pALP5 into pALP2 (E1A 13S in a pUC19-based vector) with PshAI and NdeI to create pALP7. The E1A 13S-HD2 sequence was back-cloned into pCF113 (E1A 13S in pCDNA3) to give pALP8, a pCDNA3 vector expressing the E1A 13S-HD2 fusion protein.

### Adenovirus mutagenesis

The parental virus is adenovirus 5 (ATCC VR5). The HindIII/EcoRI fragment of pUC19 was inserted into pCDNA3 to give pCF357. The region containing the Tcf-E1A and the Tcf-E1B promoters was isolated from vpCF2 [the YAC-BAC used to create vCF22, ref [12]] with KpnI and SpeI and inserted into pCF357 to create pCF358. The SphI fragment of pCF358 was inserted into pCF34 [12] to give pCF389. The region encompassing the C-terminus of E1A fused to the HD2 domain of BCL-9 was isolated from pALP8 with BsmBI and HpaI and cloned into pCF358 to create pCF387. This was cut by KpnI and XhoI and inserted into pCF389 to give pCF393. The gap repair vector was created by transferring the PacI fragment of pCF393 into pCF1 [12] to give pCF398. This plasmid contains the right and left ends of the virus separated by a SalI site. The Tcf-HD2 viral genomic plasmids (vpCF19 and vpCF21) were created by gap repair of pCF398 with genomic DNA from vKH1 and vCF22, respectively [12-14]. vpCF19 and vpCF21 were cut with PacI and converted into the viruses vCF192 and vCF212 using cR1 cells for the initial transfection and SW480 cells for the subsequent amplification and plaque purification. After expansion on SW480, the viruses were purified by CsCl banding and size exclusion chromatography (Microspin S400HR columns, Amersham, Little Chalfont, UK). Virus was stored at -70°C in 1 M NaCl, 100 mM Tris-HCl pH 8.0 and 10% glycerol. Virus was titered on SW480 cells and scored at 10 days. The particle to pfu ratios were: vKH1 36, vCF192 30, vCF22 50, vCF212 33.

### Cell lines

SW480 (ATCC CCL-228), SW620 (ATCC CCL-227), HCT116 (CCL-247) and HT29 (HTB-38) were supplied by ATCC. ISREC-O1 [15] and Co115 cells [16] were provided by Dr B Sordat. Her911 [17] and HeLa cells were provided by Dr P Beard and Dr D Lane, respectively. cR1 cells are C7 cells expressing myc-tagged ΔN-β-catenin [12]. Cell lines were grown in Dulbecco's modified Eagle's medium (DMEM) with 10% fetal calf serum (Invitrogen, Carlsbad, CA).

### Luciferase assays

The E2 reporters were described by Brunori et al [11]. They contain a minimal adenoviral E2 promoter controlled by four Tcf sites (the same promoter as is present in the vCF22 virus). Cells were seeded at 2.5 × 10^5 ^cells per 35-mm well 24 h before transfection. 100 ng of reporter plasmid and 500 ng of the vector expressing the E1A proteins or ΔN-β-catenin were used per well, and pCDNA3 empty vector was added to adjust the total amount of DNA to 1.1 μg. The DNA was incubated for 20 min with 3 μl of Lipofectamine 2000 (Invitrogen) prior to addition to cells. Cells were harvested 48 h after transfection and luciferase reporter assays were performed according to the manufacturer's instructions (Promega, Madison, WI) using a Biocounter (Lumac, Landgraaf, The Netherlands). Error bars are SD.

### Western blotting

Cells were infected with 10 plaque-forming units (pfu) per cell and harvested 24 h later in SDS-PAGE sample buffer. E1A, β-catenin, DBP, Fiber and pRb were detected with the M58 (BD Biosciences, San Diego, CA), C2206 (Sigma-Aldrich, Buchs, Switzerland), B6 [18], 4D2 (Research Diagnostics, Flanders, NJ) and C-15 (Santa Cruz Biotechnology, Santa Cruz, CA) antibodies, respectively. E4orf6 and E4orf6/7 were detected with the RSA3 antibody [19].

### Cytopathic effect and MTT assays

For CPE assays, cells in six-well plates were infected with 10-fold dilutions of virus. After 6 to 9 days, the cells were fixed with 4% formaldehyde in PBS and stained with crystal violet. For MTT assays, cells in 96-well plates were infected with 3.3-fold dilutions of virus. After 5 to 8 days, 10 μl of 5 mg/ml MTT (thiazolyl blue 98%, Sigma) was added to the medium for 3 hours. The medium was removed and the wells were dried at 37°C for 30 minutes. 100 μl of isopropanol was added to each well and the plates were shaken for 30 minutes. The absorbance was read at 562 nm on a Multiskan Ascent microplate photometer (Thermo Electron corp., Waltham, MA). Error bars are SD.

### Burst assays

Cells were infected with 1 pfu/cell. Medium was replaced 4 hours later. Cells were harvested 48 hours after infection, freeze-thawed three times and the titer was measured on HER911 cells stably expressing a Lef1-VP16 fusion protein. Error bars are SEM.

### Immunofluorescence

Cells were infected with 10 pfu/cell. 24 hours later, the wells were washed twice with PBS and the cells were fixed with 200 μl of formaldehyde 4% in PBS for 15 min. Cells were washed once with PBS and permeabilized with 0.1% Triton X-100, 1% BSA in PBS for 10 min. Cells were washed three times with PBS and blocked with 5% BSA, 5% FCS in PBS for 30 min. Cells were washed once with PBS and incubated with 200 μl of rabbit anti β-catenin antibody (100 μg/ml, C2206, Sigma) or mouse anti E1A antibody (2 μg/ml, M58, BD Biosciences) in 0.5% BSA, 0.5% FCS in DMEM (staining buffer) at 37°C for 90 min. Cells were washed three times with PBS and incubated with 200 μl of Alexa488-conjugated goat anti-mouse (20 μg/ml, a-11029, Molecular Probes, Eugene, OR) or Cy3-coupled donkey anti-rabbit (2 μg/ml, 711-165-152, Jackson Immunoresearch, West Grove, PA) secondary antibodies in staining buffer for 30 min at 37°C. Cells were washed three times with PBS. Pictures were taken with an LSM510 confocal microscope (Zeiss, Oberkochen, Germany).

### Immunoprecipitation

Cells were infected with 10 pfu/cell. 24 hours later, the cells were washed three times with ice-cold PBS pH 8.1 and lysed in 1 ml of 0.1% NP-40 buffer containing protease inhibitors. The cell extracts were collected, incubated at 4°C for 15 min and centrifuged at 13,000 rpm for 15 min at 4°C. The supernatant was collected and the protein content was measured by BCA assay (Pierce Biotechnology, Rockford, IL). One mg of total protein extract was diluted in 1 ml with PBS pH 8.1 + 0.1% BSA (solution A) and pre-cleared for 2 hours at 4°C on 100 μl of washed Dynabeads (Dynal Biotech, Oslo, Norway). One ml of a solution containing 5 μg of mouse anti-E1A antibody (M58, BD Biosciences) in solution A was incubated with washed Dynabeads. After 8 hours at 4°C, the supernatant was removed and the beads were washed three times with solution A. The pre-cleared lysate was incubated with the antibody-coupled beads overnight at 4°C, washed three times in NP-40 lysis buffer and extracted with 60 μl of SDS-PAGE sample buffer.

### Xenograft experiments

Four week old female NMRI nu/nu mice were purchased from Elevage Janvier (Le Genest St Isle, France). Subcutaneous SW620 flank xenografts were made by injecting 10^7 ^cells. Mice (six per group) were injected with virus when tumors reached 80–150 mm^3 ^in size. A total of 1 × 10^11 ^particles were injected into the tail vein, given as one shot of 1 × 10^10 ^then three shots of 3 × 10^10 ^particles at four hour intervals on day 0. This fractionated schedule was used to decrease side effects and prolong the half-life of the virus in the circulation [14]. Tumor size was measured every two days. Tumor volume was calculated according to the formula, volume = length × width^2 ^× 3.14/6. Error bars are SEM.

## Results

### E1A-HD2 transactivates Tcf-regulated promoters

**Figure 1:**
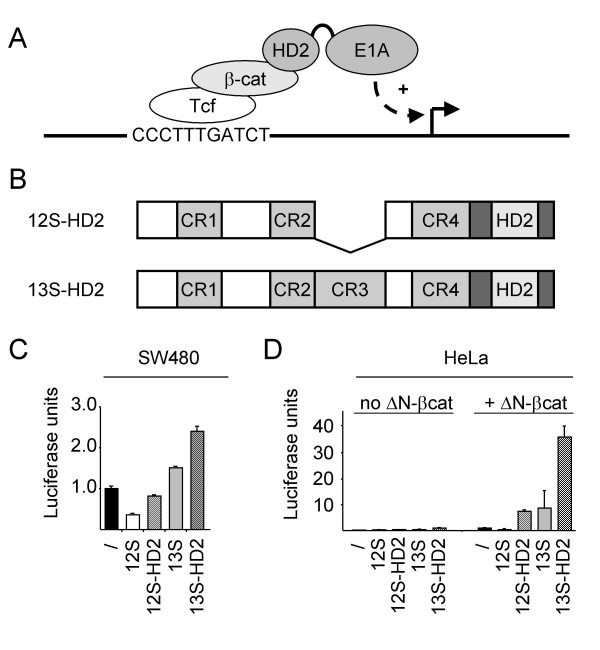
**Transcription activation by E1A-HD2 fusion proteins**. A. Diagram showing the proteins that recruit the E1A-HD2 fusion protein to Tcf sites. B. Diagram showing the fusion proteins used in the reporter assay. CR1-4: E1A conserved regions 1 to 4 [39] C. Luciferase assays in SW480 cells transfected with a Tcf-E2 reporter and E1A expression vectors. D. Luciferase assays in HeLa cells transfected with a Tcf-E2 reporter and vectors expressing E1A and N-β-catenin. The activity is expressed in arbitrary units relative to the reporter alone in C, and the reporter plus N-β-catenin in D.

We reasoned that fusion of the HD2 domain of BCL9 to E1A should lead to recruitment of E1A to Tcf-regulated promoters in cells containing stabilized β-catenin (Figure 1A). In the absence of Wnt ligand or activating mutations in the pathway, β-catenin is rapidly degraded and should be unable to recruit the fusion protein to the Tcf sites in the promoter. The HD2 domain was fused to both 12S and 13S E1A cDNAs in order to assess the contribution of the E1A CR3 domain (Figure 1B). Luciferase assays were performed using a Tcf-E2 promoter reporter [11] in SW480 cells, which have an APC mutation that activates the Wnt signaling pathway, and in HeLa cells, which lack Wnt pathway activation. As shown previously, 12S E1A repressed the promoter in SW480 cells, probably because of p300 sequestration by E1A [12]. Fusion of HD2 to 12S relieved this repression (Figure 1C). The 13S E1A protein alone had little effect, and the 13S-HD2 fusion protein activated the promoter 2.5-fold (Figure 1C). In HeLa cells, the Tcf-E2 promoter had low activity which was not affected by E1A or the fusion proteins. Cotransfection of a stabilized form of β-catenin (ΔN-β-catenin) weakly activated the promoter. This β-catenin-dependent activity was decreased by 12S E1A and increased by 12S-HD2 (Figure 1D). Cotransfection of ΔN-β-catenin and 13S E1A activated the reporter to a similar level as 12S-HD2, whereas cotransfection of ΔN-β-catenin and 13S-HD2 strongly activated the promoter (Figure 1D). Taken together, these results show that addition of the HD2 domain to E1A relieves the repression of β-catenin-dependent transcription by 12S E1A and stimulates the activation of β-catenin-dependent transcription by 13S E1A.

### E1A-HD2 binds to and promotes nuclear localization of β-catenin

**Figure 2:**
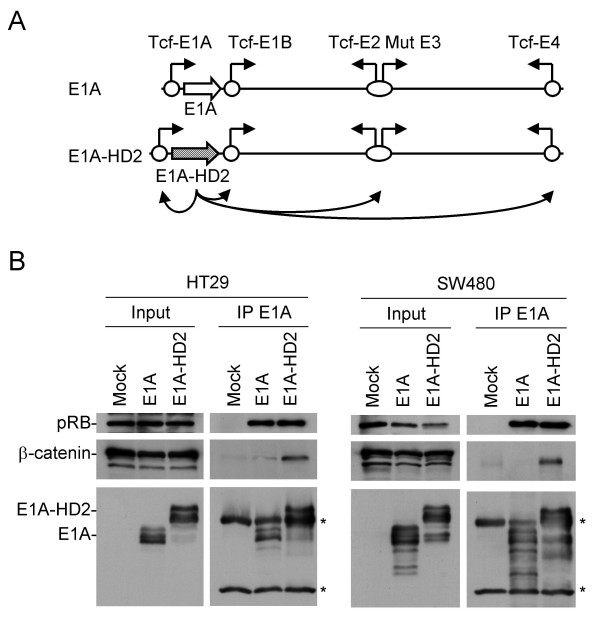
**β-catenin binds to the E1A-HD2 fusion protein in vitro**. A. Diagram showing the viruses used in this study. Open circles represent four Tcf binding sites in the viral promoters. B. Co-precipitation of β-catenin through the HD2 domain. HT29 and SW480 cells were infected with the parental and E1A-HD2 viruses. E1A immunoprecipitates were immunoblotted for β-catenin, pRb and E1A. Asterisks indicate immunoglobulin light and heavy chains.

To test the role of the E1A-HD2 fusion protein during viral infection, we inserted the E1A-HD2 gene into an oncolytic adenovirus (serotype 5) with Tcf-binding sites in the E1A, E1B, E2 and E4 promoters and deletion of the NF1, NFκB and AP1 sites in the E3 promoter [12] (Figure 2A). Co-immunoprecipitation experiments were performed to determine whether E1A-HD2 fusion proteins are able to bind to β-catenin in SW480 and HT29 cells, in which β-catenin is stabilized by mutations in APC. The cells were either mock infected or infected with the parental or E1A-HD2 viruses. Immunoblotting of cell lysates showed that E1A migrates as a larger protein after fusion to HD2. Multiple bands were seen, consistent with splicing of E1A into different isoforms including 12S and 13S (Figure 2B). Cell lysates were immunoprecipitated with an antibody against E1A and immunoblotted for β-catenin and pRb. pRb, which binds to the CR2 domain of E1A [20], was used as a positive control for the immunoprecipitation. β-catenin was co-precipitated by the E1A-HD2 fusion proteins but not by wild-type E1A proteins (Figure 2B), confirming the previous report mapping the β-catenin binding site in BCL9 to the HD2 domain [4].

**Figure 3:**
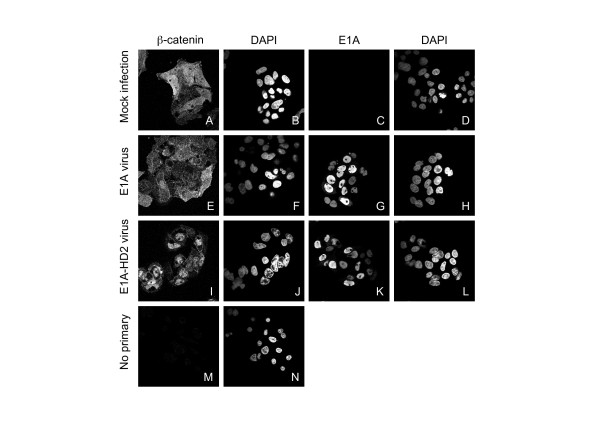
**E1A-HD2 expression promotes nuclear localization of β-catenin**. SW480 cells were mock infected (A-D), infected with the parental virus (E-H), or infected with the E1A-HD2 virus (I-L), and stained for β-catenin (A, E, I) or E1A (C, G, K). DAPI staining was used to identify the nuclei (B, D, F, H, J, L). The primary antibody was omitted in M to verify the specificity of the secondary antibody used to detect β-catenin.

To test the effect of the fusion protein on β-catenin localization, SW480 cells were infected with the parental and E1A-HD2 viruses. E1A has a strong nuclear localization signal (NLS) [21], and its nuclear localization was not altered by fusion to the HD2 domain (Figure 3G &3K). In SW480 cells, β-catenin is found at multiple sites, including the adherens junctions, cytoplasm and nucleus [Figure 3A, and ref [22]]. The localization of the protein remained unchanged when the cells were infected with the parental Tcf-regulated adenovirus (Figure 3E) but the nuclear pool of β-catenin was substantially enriched upon infection with the E1A-HD2 virus (Figure 3I). Negative controls confirmed that the E1A and β-catenin antibodies were specific (Figure 3C, mock infection; Figure 3M, no primary β-catenin antibody). We conclude that the E1A-HD2 protein binds to β-catenin and translocates β-catenin to the nucleus.

### E1A-HD2 expression increases viral protein expression and cytopathic effect

**Figure 4:**
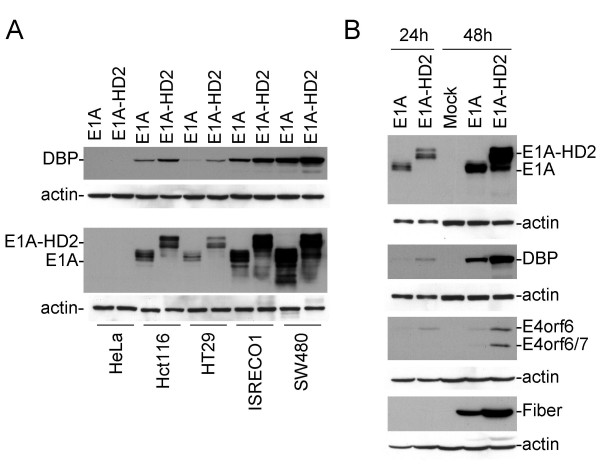
**The E1A-HD2 virus gives increased expression of viral proteins**. A. Western blot of extracts of the indicated cell lines probed for E1A and DBP (DNA Binding Protein, an E2 gene product) 24 hours after infection with the parental and E1A-HD2 viruses. B. Western blot of HT29 extracts probed for E1A, DBP, E4orf6, E4orf6/7 and fiber at 24 and 48 hours after infection with the parental and E1A-HD2 viruses.

To determine whether the E1A-HD2 protein increases the activity of the Tcf-regulated promoters in the virus, lysates of cells infected with the parental and E1A-HD2 viruses were immunoblotted for DBP and E1A 24 hours after infection. DBP is expressed from the E2 promoter, which contains four Tcf sites in both viruses. Four colorectal cancer cell lines with an activated Wnt signaling pathway (Hct116, HT29, ISREC-O1 and SW480) and one Wnt-negative cell line (HeLa) were tested. There was little effect on E1A level but a reproducible increase in the expression of DBP in all of the colon cancer cell lines infected with the E1A-HD2 virus (Figure 4A). Viral protein expression was examined in more detail in HT29 cells, which have the lowest Tcf activity of the colon cancer cell lines tested [23]. At 24 hours post infection, weak DBP and E4 protein expression were detectable, but only with the E1A-HD2 virus (Figure 4B). At 48 hours, E1A, DBP and E4 proteins were all clearly expressed at higher levels by the E1A-HD2 virus. Late protein expression, measured by immunoblotting for the fiber protein, was also higher at 48 hours, reflecting increased viral replication (Figure 4B).

**Figure 5:**
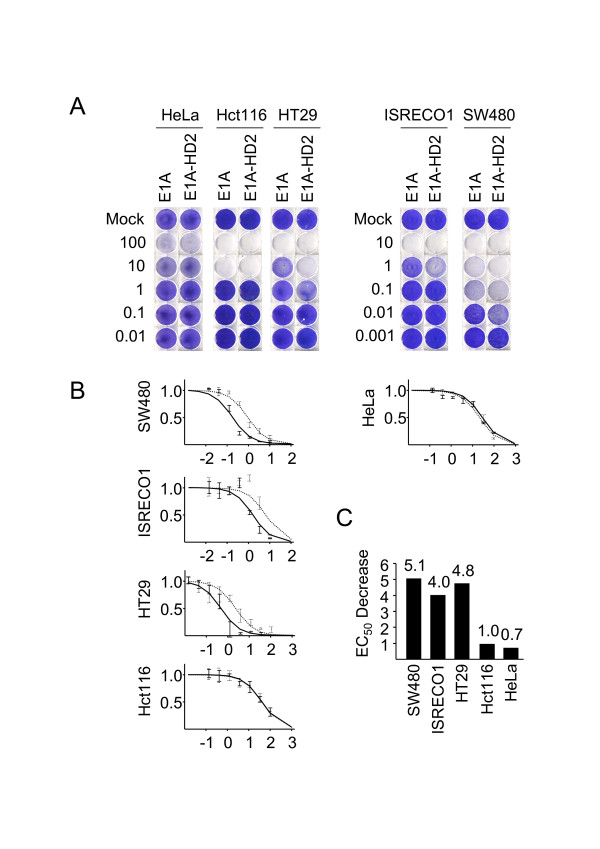
**The E1A-HD2 virus shows increased toxicity on cells with activated Wnt signaling**. A. CPE assays. Cells were infected with 10-fold dilutions of virus starting from 100 pfu/cell in HeLa, Hct116 and HT29 cells, and starting from 10 pfu/cell in ISREC-O1 and SW480 cells. B. MTT assays in the same cell lines. Dotted line, parental virus. Solid line, E1A-HD2 virus. C. The change in EC50 calculated from the data in B.

Cytopathic effect (CPE) assays were performed to determine whether increased viral protein expression led to enhanced cell killing (Figure 5A). In HeLa and Hct116 cells, the cytopathic effect of the virus was unchanged by the presence of the HD2 domain. Since the fusion protein is able to bind to β-catenin (data not shown), the lack of effect in Hct116 suggests that factors downstream of promoter activation are limiting in this colon cancer cell line. All of the remaining cell lines showed an increase in cytopathic effect (Figure 5A). MTT cell viability assays were performed to provide a quantitative measure of the increase in CPE. In HeLa and Hct116 cells, both viruses showed equivalent cell killing, whereas the E1A-HD2 virus was more toxic than the parental virus in the remaining cell lines (Figure 5B). The EC_50 _decreased five-fold in these cell lines (Figure 5C). In conclusion, the Tcf-regulated promoters were more active in colon cancer cells infected with the E1A-HD2 virus, and in most cases this resulted in a five-fold increase in cytopathic effect.

### E1A-HD2 does not increase anti-tumor efficacy in SW620 xenografts

**Figure 6:**
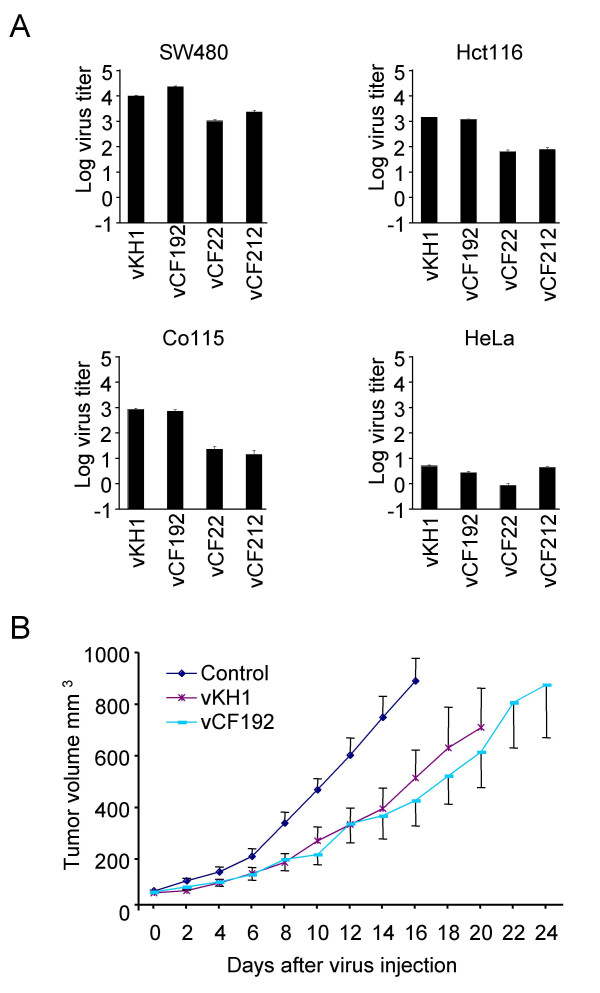
**Burst and xenograft assays**. A. Burst assays showing that the E1A-HD2 viruses vCF212 (derived from vCF22: Tcf-E1A, E1B, E2, E4) and vCF192 (derived from vKH1: Tcf-E1A, E1B, E4) have similar burst size to the parental viruses in SW480, Hct116 and Co115 colon cancer cells and HeLa cells. Error bars: SEM, n = 3. B. Growth curves of subcutaneous SW620 xenografts in NMRI nu/nu mice. Mice received 10^11 ^particles of vKH1, vCF192 or buffer control i.v. on day 0. Error bars: SEM, n = 6.

To test whether the increased pool of nuclear β-catenin and CPE translate into an increase in efficacy in vivo, we created a simpler version of the virus in which the E2 promoter is wild type (vCF192). The reason for making this choice was that the E2 promoter is the most sensitive to the level of Tcf activity in the cell. Only the E1A, E1B and E4 promoters contain Tcf sites. The vCF192 virus is identical to vKH1 [14] apart from the presence of the E1A-HD2 fusion gene. The burst size of all the viruses was tested on three colon cancer cell lines and HeLa cells (Figure 6A). There were only minimal differences between the parental viruses (vCF22 and vKH1) and the E1A-HD2 derivatives (vCF212 and vCF192). The in vivo activity of vCF192 was compared with that of vKH1 after intravenous administration of 1 × 10^11 ^particles of virus to mice with subcutaneous SW620 xenografts. SW620 colon cancer cells are derived from the same tumor as SW480 cells, and have similar high Tcf activity. We previously characterized this model and showed that virus alone delays tumor growth by about 10 days [14]. The E1A-HD2 virus (vCF192) was indistinguishable from the parental virus in this model (Figure 6B). We conclude that the small increase in cytopathic effect seen in vitro does not substantially increase the activity of the virus in cells with high Tcf activity in vivo.

## Discussion

The main conclusion from this study is that fusion of E1A to the BCL9 HD2 domain increases the activity of Tcf-regulated oncolytic adenoviruses in most colon cancer cell lines in vitro. The fusion protein binds to β-catenin, recruits it to the nucleus and increases the transcriptional activity of Tcf-regulated promoters. Despite this evidence for increased activity in vitro, the fusion protein does not increase the activity of the viruses in an SW620 xenograft model in vivo.

We and others have reported that E1A can inhibit Tcf-dependent transcription through sequestration of p300 [12,24,25]. This is not a desirable property in a Tcf-regulated adenovirus because it interferes with strong activation of the promoters. We previously attempted to circumvent this problem by mutating the p300 binding site in E1A, but the resulting viruses were 10-fold attenuated relative to matched viruses with wild type E1A, indicating that E1A-binding to p300 is more important for other functions of the virus than inhibition of cellular Tcf-dependent transcription [12]. We report here an alternative solution to the problem: fusion of HD2 to the C-terminus of E1A relieves the repression of Tcf-regulated transcription by E1A. Unlike mutation of the p300 binding site, the resulting viruses have increased activity. The relief of transcriptional repression by E1A can not be explained by concurrent transcriptional activation through pygopus, unless HD2 domains can form mixed oligomers that allow simultaneous recruitment of E1A-HD2 and BCL9. An alternative explanation is that 12S E1A-HD2 recruits E1A-p300 complexes which retain some p300 functions that contribute positively to transcriptional activation [26].

Low Tcf activity has been reported in about half of the colon cancer cell lines where it has been tested, despite the presence of confirmed mutations in either APC or β-catenin [23]. One reason for the low activity is thought to be inadequate localization of β-catenin to the nucleus. β-catenin exists in three pools, a stable pool bound to E-cadherin in adherens junctions, and unstable cytosolic and nuclear pools. Loss of E-cadherin has been reported to increase Tcf activity in some systems [27], but the major factor responsible for the Tcf activation in tumors is the stabilization of β-catenin, which increases the size of the cytosolic and nuclear pools of β-catenin. The relative abundance of the two pools depends on the balance of import and export of β-catenin from the nucleus [28]. β-catenin itself lacks import or export signals, but can interact directly with nuclear pore proteins [29] and may be imported in a complex with BCL9 [30]. APC has been shown to mediate export of β-catenin through clusters of nuclear export signals (NES) at the N-terminus and in the central region preceding the axin binding site [31,32]. APC mutations in tumors predominantly truncate the protein before the axin binding site, which is important for stabilization of β-catenin, but occasionally retain the central export signals. Cell lines like HT29, which contain APC mutants that retain the ability to export β-catenin, tend to have lower Tcf activity than cell lines like SW480, which truncate APC before the central NES cluster [23]. Retention of the central NES cluster is rare when APC is mutant, but retention of wild type APC is common in tumors from patients with HNPCC. Consistent with the ability of APC to export β-catenin from the nucleus, silencing of APC expression by RNAi has been shown to increase the Tcf activity of an HNPCC cell line with mutant β-catenin and wild type APC [33]. Taken together, these data suggest that promoting nuclear localization of β-catenin by fusion of HD2 to E1A, which has a strong NLS, might increase the activity of the E1A-HD2 viruses. It is important to note, however, that there are many cell lines where low Tcf activity is at best only partially explained by differences in nuclear import and export of β-catenin. Co-expression of BCL9 and pygopus promoted nuclear localization of β-catenin in SW480 but failed to do so in 293T, HT29 and Hct116 cells [30], and silencing of APC by RNAi only leads to Tcf activation in a minority of cases (CF and RI, unpublished data). There are certainly many other factors which can interfere with β-catenin transport or Tcf activity, such as tyrosine phosphorylation or binding to ICAT or chibby [34-37].

The E1A-HD2 gene is expressed from a Tcf-regulated promoter in the HD2 virus. This should result in positive feedback regulation of E1A-HD2 expression. E1A-HD2 expression by the new virus was clearly higher than E1A expression by the parental virus in HT29 cells at 48 hours, but the difference at 24 hours in HT29 and the other colon cancer cell lines was small. There was a bigger effect on expression of the E2 and E4 proteins, suggesting that recruitment of E1A to the other early promoters is more important than positive feedback of E1A on its own expression.

The E1A-HD2 virus was more active in CPE assays in SW480, ISRECO1 and HT29 but not Hct116 cells. The reason for the lack of effect on CPE in Hct116 is unknown. Hct116 cells have a mutation in p300, which may limit the ability of E1A-HD2 to activate the viral promoters [38], although it seems unlikely that this is the only explanation because DBP was expressed better by the E1A-HD2 virus than the parental Tcf-regulated virus (Figure 4A). Hct116 cells are deficient in mismatch repair and consequently contain point and insertion/deletion mutations scattered throughout the genome, some of which may limit the activity of the virus downstream of early viral promoter activation. To test the fusion strategy in vivo we made a simpler version of the virus (vCF192) with a wild type E2 promoter because the virus with Tcf sites in all the early promoters is too attenuated to be useful in vivo. The resulting E1A-HD2 virus was indistinguishable from the parental virus in the xenograft model we tested. It is possible that xenografted cell lines with lower Tcf activity would show a preference for the E1A-HD2 virus in vivo, but this was not pursued because of the relatively small difference in activity of the E1A-HD2 viruses in vitro. Since the block in cells with low Tcf activity is refractory to increases in the nuclear β-catenin pool, it seems likely that a more fruitful oncolytic strategy would be to make viruses that target steps downstream of β-catenin.

**Figure 7:**
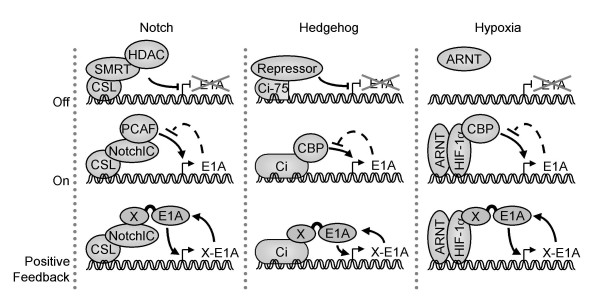
**Three possible examples of tumor-specific targeting using E1A fusion proteins**. In each case E1A is fused to a protein domain (X) that binds to a protein normally found at the promoter in increased amounts in tumor cells. Notch Pathway: in the absence of a signal, CSL recruits co-repressors; upon signaling, NotchIC is released, binds to CSL and activates transcription. Hedgehog pathway: in the absence of a signal, Cubitus Interruptus is cleaved to its repressor form; upon signaling, full-length Ci is produced and activates transcription. Hypoxia pathway: in normoxia, HIF1α is degraded; in hypoxia, HIF1α is stabilized, dimerizes with ARNT, binds to DNA and activates transcription.

In principle, our fusion protein strategy could be used to increase the activity of all types of oncolytic viruses, without loss of selectivity for tumor cells. The general argument for an adenovirus would be to place binding sites for a protein Y in the early viral promoters and fuse E1A to a protein X that can bind to Y. This would trigger a positive feedback loop in cells with Y at the viral promoters. Tumor selectivity depends on the fusion protein being unable to activate transcription in a normal cell, which means that Y should not be present at the viral promoters in these cells. We show three examples of pathways that could be targeted using this approach in Figure 7. In each case, the critical change is that the amount of Y at the promoter is greater in tumor cells, either because of oncogenic mutations or tumor hypoxia.

## Conclusion

Fusion of the BCL9 HD2 domain to E1A increased the nuclear pool of β-catenin, consistent with the presence of a strong NLS in E1A, but this resulted in only small changes in the activity of the virus. This indicates that the amount of β-catenin in the nucleus is not limiting for viral activity in colon cancer cell lines with low Tcf activity, and suggests that factors downstream of β-catenin are limiting for viral toxicity in these cells.

## Abbreviations

12S/13S, Alternatively spliced transcripts of E1A

APC, Adenomatous Polyposis Coli

ATF, Activating Transcription Factor

BCL9, B Cell Lymphoma 9

BSA, Bovine Serum Albumin

CPE, Cytopathic Effect

CR3, E1A Conserved Region 3

DBP, Adenoviral DNA Binding Protein

E1A/E1B/E2/E4, Adenoviral early transcription units

HD2, Homology Domain 2

HNPCC, Hereditary Non-Polyposis Colon Cancer

ICAT, Inhibitor of β-catenin

MTT, 3-(4,5-Dimethylthiazol-2-yl)-2,5-diphenyltetrazolium bromide

NES, Nuclear Export Signal

NHD, N-terminal Homology Domain

NLS, Nuclear Localization Signal

PBS, Phosphate Buffered Saline

Tcf/LEF, T cell factor/Lymphoid Enhancer Factor

Wnt, Wingless Integration site

YAC-BAC, Yeast Artificial Chromosome-Bacterial Artificial Chromosome

## Competing interests

The author(s) declare that they have no competing interests.

## Authors' contributions

CF conceived the study, produced and characterized vCF212, and helped to draft the manuscript. KH produced and characterized vCF192. ANL performed the animal experiments. ALP constructed the pALP plasmids and performed the luciferase assays. RDI supervised the lab, participated in the design of the study and wrote the manuscript. All authors read and approved the final manuscript.

## Pre-publication history

The pre-publication history for this paper can be accessed here:
